# KCTD11 inhibits growth and metastasis of hepatocellular carcinoma through activating Hippo signaling

**DOI:** 10.18632/oncotarget.17145

**Published:** 2017-04-17

**Authors:** Rongliang Tong, Beng Yang, Heng Xiao, Chuanhui Peng, Wendi Hu, Xiaoyu Weng, Shaobing Cheng, Chengli Du, Zhen Lv, Chaofeng Ding, Lin Zhou, Haiyang Xie, Jian Wu, Shusen Zheng

**Affiliations:** ^1^ Department of Surgery, Division of Hepatobiliary and Pancreatic Surgery, First Affiliated Hospital, School of Medicine, Zhejiang University, Hangzhou 310000, China; ^2^ Key Laboratory of Combined Multi-Organ Transplantation, Ministry of Public Health, Hangzhou 310000, China; ^3^ Key Laboratory of Organ Transplantation, Zhejiang Province, Hangzhou 310000, China; ^4^ Department of Hepatobiliary Surgery, First Affiliated Hospital, Chongqing Medical University, Chongqing 400016, China; ^5^ The Collaborative Innovation Center for Diagnosis and Treatment of Infectious Diseases, Zhejiang University, Hangzhou 310000, China

**Keywords:** KCTD11, hepatocellular carcinoma, Hippo pathway, p21, cell adhesion

## Abstract

A lack of effective prognostic biomarkers and molecular targets is a serious problem in hepatocellular carcinoma. KCTD11, reported as a tumor suppressor, are still not well understood. In this study, KCTD11 was found low-expressed in HCC tissues and cell lines. The HCC patients with low expression of KCTD11 suggested shorter overall survival. We found KCTD11 inhibiting cell proliferation *in vitro* and tumor growth *in vivo*, by activating p21 and repressing cycle related proteins. KCTD11 also inhibited cell adhesion by decreasing CTGF and CLDN1. We found CTGF binding COL3A1 in HCCLM3, which might lead to reduction of COL3A1 expression. KCTD11 also inhibited cell migration and invasion in HCC, by repressing MMPs and EMT. We found the tumor suppression function of KCTD11 was at least partly through activating Hippo pathway in HCC. Base on the enhanced Hippo pathway, KCTD11 could activate p21 by stabilizing p53 or promoting the MST1/ GSK3β/p21 signaling in HCC. Overall, these results suggest that KCTD11 works as a tumor suppressor and owns prognostic and therapeutic potentials in HCC.

## INTRODUCTION

The number of new liver cancer cases and deaths worldwide are both estimated over 700,000 per year [1]. In these cases, hepatocellular carcinoma (HCC) makes up the majority. The prognosis of HCC patients is still poor. Personalized therapy of HCC is in short of effective molecular targeted drug [2, 3]. All in all, novel biomarkers and molecule targets are critically-needed in predicting, diagnosing and treating HCC [4].

KCTD11 is identified as a tumor suppressor gene antagonizing Hedgehog signaling in human medulloblastoma (MB) [5, 6]. Decreased expression of KCTD11 was found in some tumors such as human MB and prostate adenocarcinoma. Loss of heterozygosity (LOH), Sp1 transcription factor and DNA methylation seems to be the reasons of dysregulation of KCTD11 [6–8]. As is reported, the biological processes regulated by KCTD11 contains cell proliferation and apoptosis [6, 9]. However, the role of KCTD11 in HCC remains unclear.

In this study, KCTD11 is found inhibiting cell growth and metastasis of HCC. The tumor suppression function of KCTD11 is at least partly in a way of activating Hippo pathway. Overall, we present a novel mechanism and a novel molecule with prognostic and therapeutic potentials in HCC.

## RESULTS

### KCTD11 is low-expressed in HCC tissues

**Figure 1:**
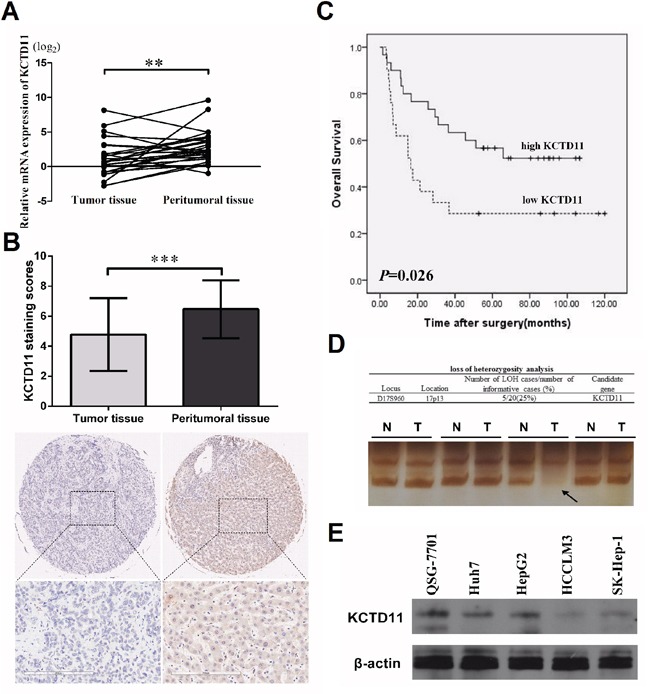
KCTD11 is low-expressed in HCC tissues **(A)** Relative mRNA expression of KCTD11 is lower in 26 pairs of HCC tissues than peritumoral tissues (Paired Student's t-test). Data of every sample is from three independent experiments. Data is expressed as mean ± SD. **(B)** Immunohistochemistry scores of a tissue microarray with 45 pairs of HCC samples are shown. Staining scores and one case are shown (Paired Student's t-test). The protein levels of KCTD11 is lower in tumor tissues. Data is expressed as mean ± SD. Bar=200μm. **(C)** Overall survival rates of the HCC patients with low- and high-expression of KCTD11 were shown (Log Rank test). **(D)** LOH analysis is performed in 20 pairs HCC samples with matching normal liver tissues. D17S960, specific microsatellite marker. Representative image of KCTD11 LOH in HCC is shown. **(E)** Western blot shows the protein expression of KCTD11 in QSG-7701and four liver cancer cell lines. Beta-actin is used as a loading control. **P<0.01, ***P<0.001.

With the hypothesis that KCTD11 is a tumor suppressor in HCC, we start our research from evaluating the expression of KCTD11 in HCC. We examined the mRNA expression of KCTD11 in 26 pairs of HCC tissues by RT-PCR, KCTD11 was found lower expressed in tumor tissues (Figure 1A). Immunohistochemistry was then performed to detect the protein expression of KCTD11 in a commercial tissue microarray with 45 pairs of HCC tissues. Cytoplasmic and nuclear expression of KCTD11 was both found in HCC samples. The protein levels of KCTD11 was also found lower in tumor tissues than peritumoral tissues (Figure 1B). And, low expression of KCTD11 suggested shorter overall survival after surgery (Figure 1C).

To further study the reasons of KCTD11 dysregulation in HCC tissues, we performed LOH analysis in 20 HCC samples matched with paired peritumoral tissues. D17S960 microsatellite was analyzed to reveal the deletion of KCTD11 allele, KCTD11 allelic deletion was found in 5 samples (Figure 1D and data not shown).

We further compared the protein expression of KCTD11 in an immortalized normal liver cell line (QSG-7701) and four liver cancer cell lines (HepG2, Huh7, HCCLM3, SK-Hep-1) by western bolt. The expression of KCTD11 was higher in QSG-7701 than the four HCC cell lines. Lower expressed KCTD11 was found in high metastatic HCC cell lines (HCCLM3, SK-Hep-1) than low metastatic HCC cell lines (HepG2, Huh7) (Figure 1E).

In this part, a down-regulation of KCTD11, which partly caused by LOH, was shown in HCC tissues and cell lines. The aberrantly expression of KCTD11 suggested that KCTD11 may be a tumor suppress gene in HCC.

### KCTD11 inhibits cell proliferation of HCC *in vitro*

**Figure 2:**
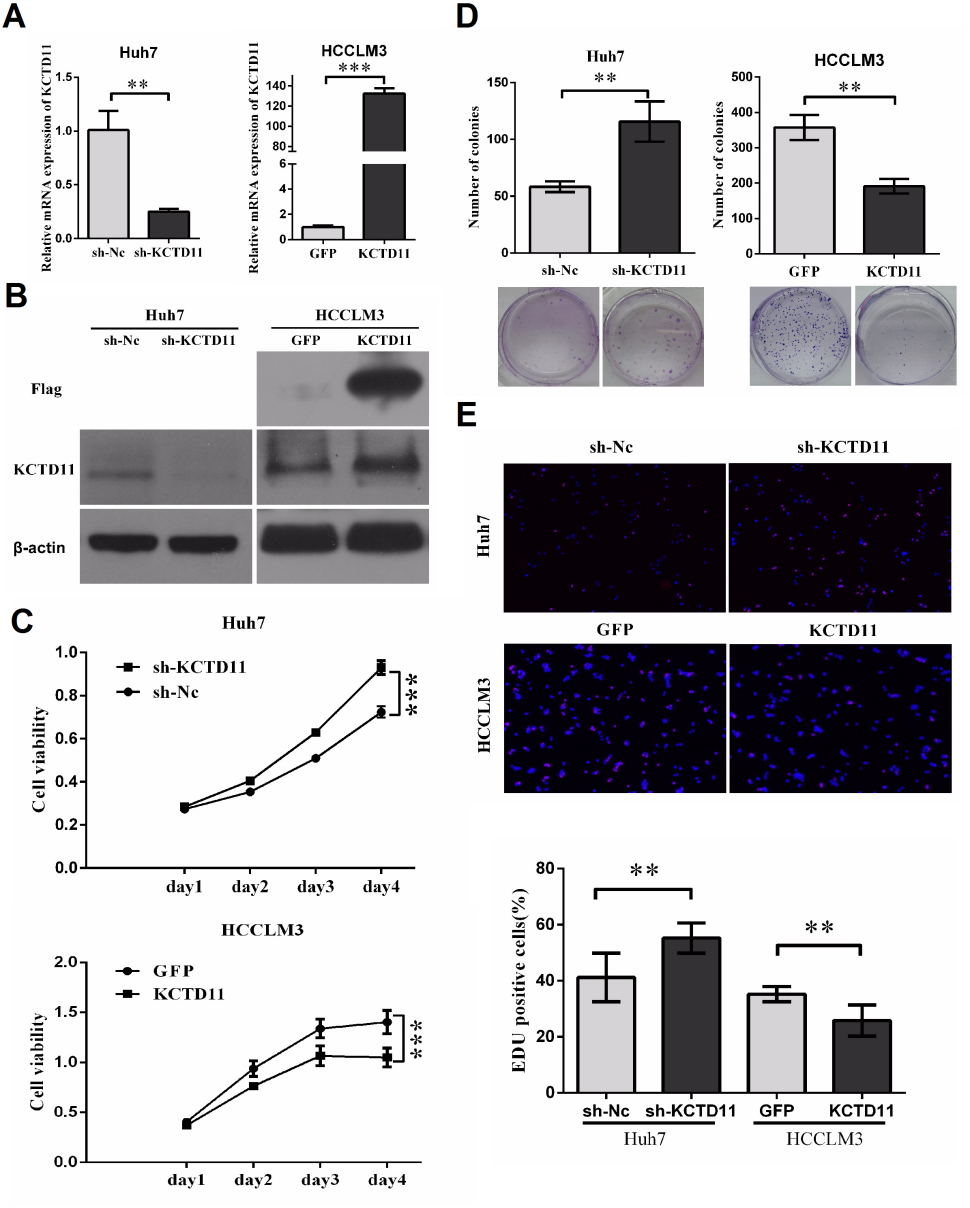
KCTD11 inhibits cell proliferation of HCC *in vitro* **(A)** With lentivirus transfected, Huh7 and HCCLM3 were evaluated KCTD11 mRNA expression by RT-PCR. Data is from three independent experiments and expressed as mean ± SD. **(B)** With lentivirus transfected, Huh7 and HCCLM3 were evaluated KCTD11 and Flag protein expression by western blot in. Beta-actin is used as a loading control. **(C)** Knock-down of KCTD11 increases cell viability in Huh7. Overexpression of KCTD11 reduces cell viability in HCCLM3. Cell viability is assayed using CCK8 assay. Data is from over three independent experiments and expressed as mean ± SD. **(D)** KCTD11 knock-down increases colony formation in Huh7. KCTD11 overexpression inhibits colony formation in HCCLM3. Representative colony images are shown. Data is from three independent experiments and expressed as mean ± SD. **(E)** KCTD11 knock-down increases EDU positive cells in Huh7. KCTD11 over-expression reduces EDU positive cells in HCCLM3. Representative images are shown. Data is from three independent experiments and expressed as mean ± SD. **P<0.01, ***P<0.001.

To investigate the function and mechanisms of KCTD11 in HCC, stably ectopic expression of KCTD11 (marked with a Flag tag) was performed in a high metastatic HCC cell line (HCCLM3), while stably knock-down was performed in a low metastatic HCC cell line (Huh7), by transfection assay with lentiviral vectors. The effect of transfection was confirmed by RT-PCR and western bolt (Figure 2A, 2B).

To research the function of KCTD11 in cell proliferation, we performed cell viability assay and colony formation assay. KCTD11 knock-down increased cell viability and promoted colony formation in Huh7. Up-regulation of KCTD11 induced inhibition of cell viability and colony formation in HCCLM3 (Figure 2C, 2D). The suppressive function of KCTD11 on cell growth was further verified in EDU assay in the two HCC cell lines. KCTD11 knock-down increased EDU positive cells in Huh7, while KCTD11 over-expression reduced EDU positive cells in HCCLM3 (Figure 2E).

The data above suggested that KCTD11 could suppress HCC cell proliferation *in vitro*.

### KCTD11 inhibits tumor growth *in vivo* and elicits a tumor inhibitory effect by increasing p21 and decreasing cycle related proteins in HCC

**Figure 3:**
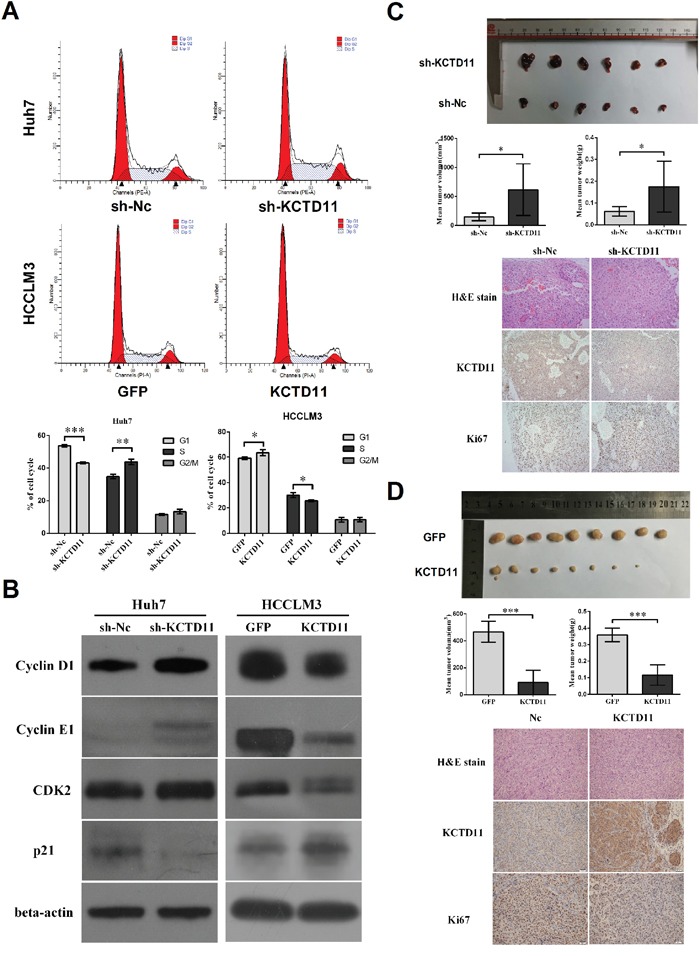
KCTD11 inhibits tumor growth *in vivo* and elicits a tumor inhibitory effect by increasing p21 and decreasing cycle related proteins in HCC **(A)** KCTD11 induces G1/S cycle arrest. Representative images are shown. Data is from three independent experiments and expressed as mean ± SD. **(B)** Expressions of p21, Cyclin D1, CDK4, CDK6, Cyclin E1 and CDK2 are detected by western blot in the transfected cells. Beta-actin is used as a loading control. **(C)** and **(D)** Volume and weight of the tumors in subcutaneous xenograft tumorigenesis model are shown. KCTD11 knock-down in Huh7 promotes tumor growth. KCTD11 overexpression in HCCLM3 results in reduction of tumor growth. Data is expressed as mean ± SD. Representative H&E staining images and IHC staining images of the tumors from subcutaneous xenograft experiment are shown. Bar=200μm. *P<0.05, **P<0.01, ***P<0.001.

In cell cycle assay, KCTD11 knock-down induced decrease of G1-phase and increase of S-phase in Huh7, and KCTD11 overexpression induced increase of G1-phase and decrease of S-phase in HCCLM3 (Figure 3A). This result suggested that KCTD11 could induce G1/S cell cycle arrest in HCC. So, cycle related proteins were then estimated by western bolt in the transfected cells. KCTD11 knock-down repressed p21 and promoted the expression of Cyclin D1, Cyclin E1 and CDK2. KCTD11 overexpression resulted in up-regulating of p21 and down-regulating of Cyclin D1, Cyclin E1 and CDK2 (Figure 3B).

In the subcutaneous xenograft tumorigenesis model base on the transfected cells, volume and weight of the tumors were measured, and immunohistochemistry was performed to estimate the expression of KCTD11 and Ki67. KCTD11 knock-down in Huh7 increased tumor volume and weight, and promoted Ki67 expression (Figure 3C). KCTD11 overexpression in HCCLM3 resulted in reduction of tumor volume and weight, and repressing Ki67 (Figure 3D).

In summary, KCTD11 induced G1/S cell cycle arrest and effectively inhibited tumorigenesis of HCC *in vivo*.

### KCTD11 inhibits cell adhesion by decreasing CTGF and CLDN1

**Figure 4:**
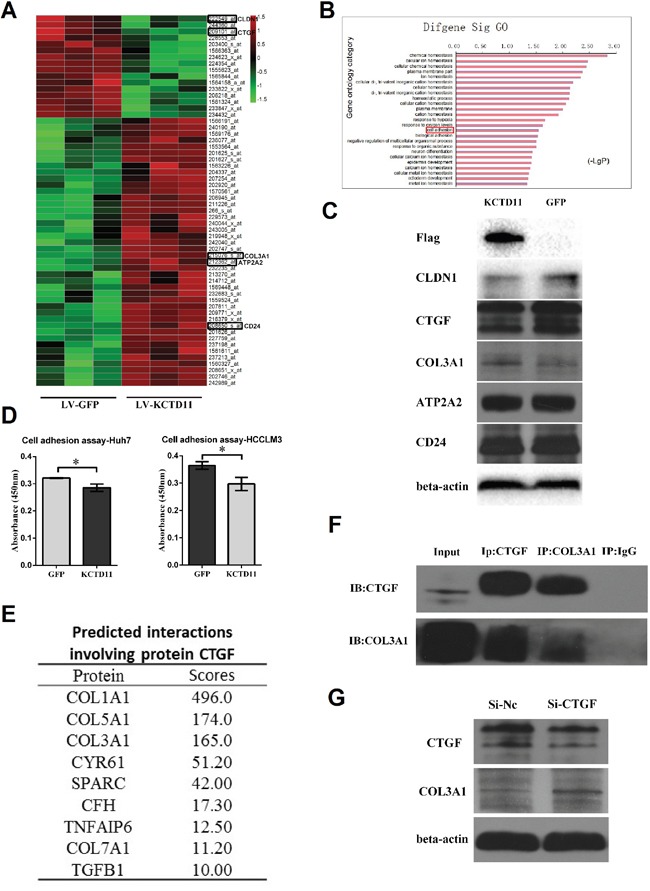
KCTD11 inhibits cell adhesion by decreasing CLDN1 and CTGF **(A)** The genome-wide gene expression affected by KCTD11 overexpression in an Affymetrix Human U133 plus 2.0 array. (Fold change > 2.0 and p < 0.05) **(B)** Gene Ontology analysis of the genes regulated by KCTD11 overexpression. **(C)** After KCTD11 up-regulated, the expressions of CLDN1, CTGF, COL3A1, ATP2A2 and CD24 are detected by western blot. Beta-actin is used as a loading control. **(D)** Overexpression of KCTD11 inhibits cell adhesion in Huh7 and HCCLM3. Data is from three independent experiments and expressed as mean ± SD. *P<0.05. **(E)** Predicted protein bindings involving protein CTGF. **(F)** Co-immunoprecipitation assay confirms the protein binding between CTGF and COL3A1. **(G)** Knockdown of CTGF results in up-regulating of COL3A1 in western blot assay. Beta-actin is used as a loading control.

To further investigate the regulation targets of KCTD11, an Affymetrix genome-wide gene expression array was used to screen the expression changes of genes in HCCLM3 cells with KCTD11 overexpression (Figure 4A). DAVID bioinformatics database was used to perform Gene Ontology analysis to analyze the genes regulated (fold change > 2.0 and p < 0.05) by KCTD11 [10, 11]. Cell adhesion and biological adhesion was found associated with the expression level of KCTD11 (Figure 4B). These genes related to adhesion contained CLDN1, CTGF, COL3A1, ATP2A2 and CD24. Then, the protein expression levels of these genes were detected in HCCLM3 cells with KCTD11 overexpression. With KCTD11 overexpressed, the protein expression of CLDN1 and CTGF decreased and those of COL3A1 increased (Figure 4C), which share the same tendency with the results of genome-wide gene expression. However, the protein expression of ATP2A2 and CD24 were not changed with KCTD11 up-regulation. Cell adhesion assay was then conducted and overexpression of KCTD11 was found resulted in inhibiting cell adhesion (Figure 4D).

As CLDN1, CTGF and COL3A1 were reported to promote cell adhesion [12–15], the up-regulation of COL3A1 after KCTD11 overexpression confused us. To solve this problem, the relationships between these genes related to adhesion were researched. A human protein-protein interactions prediction database was used to predict the interactions of these proteins [16, 17]. The protein-protein binding between CTGF and COL3A1 was suggested in this database (Figure 4E). Then, the protein binding of CTGF and COL3A1 was confirmed by co-immunoprecipitation assay in HCCLM3 (Figure 4F). To study the biological function of the binding between CTGF and COL3A1, HCCLM3 was treated by siRNA of CTGF. With the reduction of CTGF protein, the protein level of COL3A1 was up-regulated (Figure 4G). Thus, we ascribed the up-regulation of COL3A1 to the down-regulation of CTGF.

Taken together, KCTD11 inhibited cell adhesion by decreasing the expression of CTGF and CLDN1. Furthermore, we demonstrated the protein-protein binding of CTGF and COL3A1, and the binding might result in reducing COL3A1 expression.

### KCTD11 inhibits cell migration and invasion in HCC *in vitro* and inhibits tumor metastasis *in vivo*, by repressing MMPs and EMT

**Figure 5:**
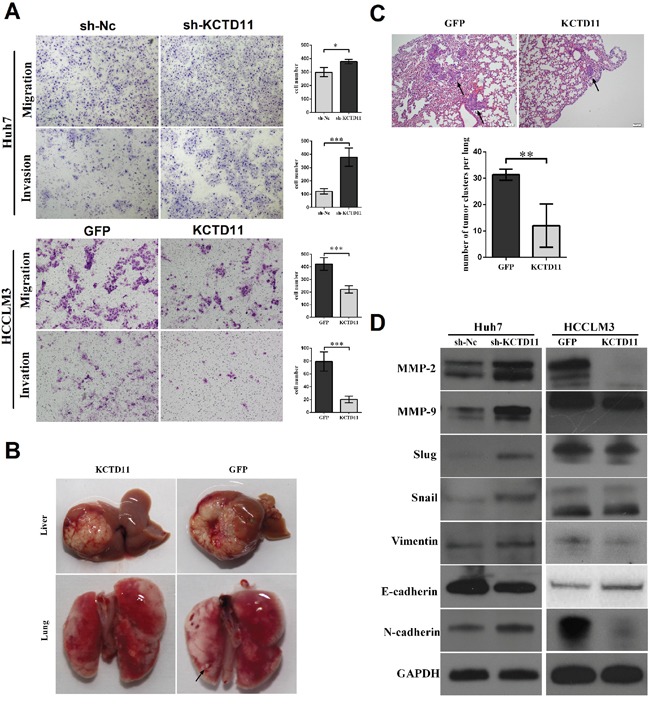
KCTD11 inhibits cell migration and invasion in HCC *in vitro* and inhibits tumor metastasis *in vivo*, by repressing MMPs and EMT **(A)** KCTD11 knock-down promotes cell migration and invasion in Huh7. KCTD11 overexpression inhibits cell migration and invasion in HCCLM3. Representative images are shown. Data is from three independent experiments and expressed as mean ± SD. **(B)** Nude mice liver orthotopic tumorigenesis experiment to evaluate lung metastasis. Representative images of the livers and lungs are shown. The tumor metastasis in lung is marked by arrows. **(C)** KCTD11 overexpression inhibits lung metastasis of HCC. The tumor clusters in lung are marked by arrows. Data are expressed as mean ± SD. **(D)** Expressions of Flag, MMP-2, MMP-9, Slug, Snail, E-cadherin, N-cadherin and Vimentin are detected by western blot. GAPDH is used as a loading control. *P<0.05, **P<0.01, ***P<0.001.

As CLDN1 and CTGF were reported to promote EMT [18, 19], we continued to research the effect of KCTD11 on EMT. The migratory and invasive capacity was promoted in Huh7 with KCTD11 knock-down, and was inhibited in HCCLM3 with KCTD11 over-expressed (Figure 5A). Then, we established an orthotopic liver tumor model to investigate the effect of KCTD11 on pulmonary metastasis (Figure 5B). In HCCLM3, KCTD11 overexpression was found inducing reduction of lung metastasis clusters (Figure 5C).

We performed western bolt to examine the expression changes of MMPs and the key proteins related to EMT in Huh7 and HCCLM3. In Huh7, KCTD11 knock-down increased the expression of MMP2, MM9, Slug, Snail, N-cadherin and Vimentin, and reduced the expression of E-cadherin. In HCCLM3, with KCTD11 up-regulated, MMP2, MM9, Slug, Snail, N-cadherin and Vimentin was repressed, and the expression of E-cadherin was promoted (Figure 5D).

In summary, KCTD11 suppressed cell migration and invasion *in vitro* and inhibits tumor metastasis *in vivo*, by repressing MMPs and EMT.

### KCTD11 activates Hippo pathway in HCC, and activates p21 by stabilizing p53 and promoting the MST1/GSK3β/p21 signaling

**Figure 6:**
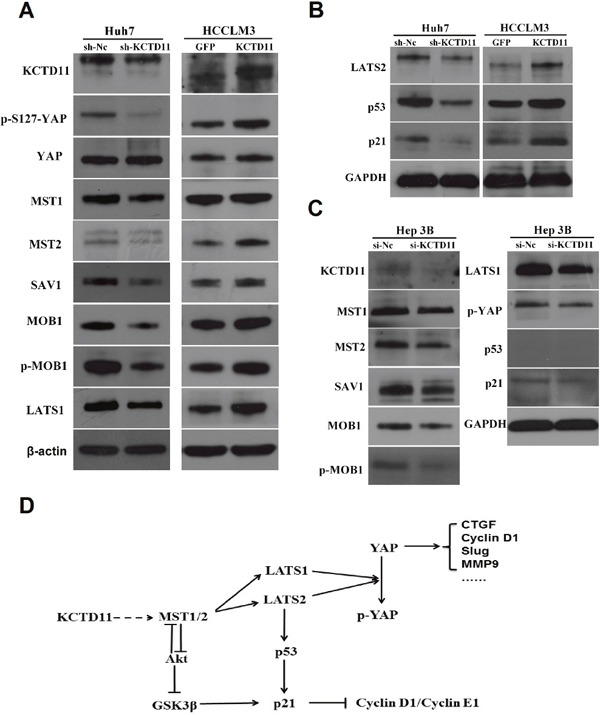
KCTD11 activates Hippo pathway in HCC, and activates p21 by stabilizing p53 and promoting the MST1/GSK3β/p21 signaling **(A)** Expressions of the key proteins in Hippo pathway are detected by western blot in Huh7 (p53 mutant) and HCCLM3 (wild p53). Beta-actin is used as a loading control. **(B)** Expressions of LATS2, p53 and p21 are detected by western blot in Huh7 (p53 mutant) and HCCLM3. GAPDH is used as a loading control. **(C)** Expressions of LATS2, p53 and p21are detected by western blot in Hep3B (p53-deficient). GAPDH is used as a loading control. **(D)** KCTD11 inhibits tumor progression in at least three different ways by activating Hippo pathway: inactivating YAP, stabilizing p53 and promoting the MST1/GSK3β/p21 signaling.

With the results above, KCTD11 was found to be regulating not a few molecules related to tumor progression, such as Cyclin D1, CTGF, MMP9 and Slug. Those suggested that the mechanism role of KCTD11 may be related to some potent signal pathways. As reported, YAP, a transcription co-activators, could transcriptional regulate Cyclin D1, CTGF, MMP9 and Slug [20, 21]. Hippo pathway could repress the function of YAP [22]. We then estimated the expression changes of Hippo pathway by western bolt in the transfected cells. Expression of MST1, MST2, SAV1, MOB1, p-MOB1, LATS1 and p-YAP (S127) decreased markedly after KCTD11 knock-down in Huh7. Expression of MST2, SAV1, MOB1, p-MOB1, LATS1 and p-YAP (S127) increased markedly after KCTD11 overexpression in HCCLM3. However, the expression of YAP was not markedly changed (Figure 6A). Those suggested that KCTD11 could activate Hippo pathway, promote the phosphorylation of YAP at Ser127, and then accordingly negatively regulate the transcriptional function of YAP.

It was reported that LATS2 could stabilizes p53 [22]. To reveal the mechanism of the enhanced p21 signaling after KCTD11 overexpression, we estimated the expression of LATS2 in the transfected cells. Expression of LATS2, p53 and p21 decreased markedly after KCTD11 knock-down in Huh7, and increased markedly after KCTD11 overexpression in HCCLM3 (Figure 6B).

However, the p53 in Huh7 was mutant and could not activate p21 [23, 24], so the repression of p21 after KCTD11 knock-down in Huh7 was still confusing. Interestingly, MST1 could negatively regulated Akt activity and thereby promote GSK3β [25, 26], which could activate p21 [27]. So, the repression of p21 after KCTD11 knock-down in Huh7 could be a result of up-regulated MST1/GSK3β. To further confirm this mechanism, we estimated the changes of Hippo pathway and p21 signaling in the p53-deficient Hep3B cells [28]. Hep3B was treated by siRNA of KCTD11. Consistent with the hypothesis above, KCTD11 knock-down in Hep3B activated Hippo pathway and also repressed p21, without the participation of p53 (Figure 6C).

Taken together, the tumor suppression function of KCTD11 was at least partly through activating Hippo pathway, which could inhibit cell proliferation in at least three ways: inactivating YAP, stabilizing p53 and promoting the MST1/GSK3β/p21 signaling (Figure 6D).

## DISCUSSION

Though the tumor suppression role of KCTD11 has been described in human MB and prostate adenocarcinoma, intensive and systematic researches are still needed to illuminate the value of this molecule in study and treatment of tumors.

In this study, the down-regulation of KCTD11 in HCC tissues and cell lines suggests KCTD11 could be a tumor suppress gene and may possess important function in normal liver cells. With the results of assays *in vitro* and vivo, we demonstrate the tumor suppressing ability of KCTD11 in HCC. KCTD11 inhibits cell proliferation *in vitro* and tumor growth *in vivo* by activating p21 and repressing cycle related proteins. KCTD11 inhibits cell adhesion, migration and invasion by inhibiting CTGF and repressing MMPs and EMT. As YAP transcriptional activates Cyclin D1, CTGF, MMP9 and Slug, we speculate KCTD11 could be correlated with Hippo pathway. Then, the western bolt results indicate that KCTD11 can activate Hippo pathway. So, we ascribe at least partly of the tumor suppression function of KCTD11 to enhanced Hippo pathway. Then, to reveal the regulating mechanism between KCTD11 and p21, we found LATS2 stabilizes p53 and thereby activates p21, while MST1 could promote GSK3β and thereby also activate p21. So we think KCTD11 inhibits cell proliferation in at least three different ways by activating Hippo pathway: inactivating YAP, stabilizing p53 and promoting the MST1/GSK3β/p21 signaling.

However, different from other tumors such as human MB and prostate adenocarcinoma, neither enhancing expression of p27^Kip1^ nor antagonizing Hedgehog signaling by regulating Gli1 [6, 29] has be found in HCC cells after KCTD11 overexpression (data not shown). The different biological mechanisms could be a result of heterogeneity of different tumors.

As an important biological process, cell adhesion could affect tumorigenesis by regulating cell-cell contact, dedifferentiation, invasiveness and microenvironment [30, 31]. In this study, the finding of genes related to cell adhesion in genome-wide gene expression array encourages us to speculate that KCTD11 may regulate cell adhesion in tumors. Then, CLDN1, CTGF and COL3A1 are defined as downstream molecules of KCTD11. It is important to point out that up-regulation of COL3A1 by KCTD11 overexpression is actually a result of CTGF reduction. And, this regulation may base on the protein-protein interaction between CTGF and COL3A1, according to the data of co-immunoprecipitation assay.

In summary, we identify KCTD11 as a tumor suppressor in HCC. KCTD11 actives Hippo pathway, and thereby up-regulate p21 and repress YAP, these result in inhibition of cell proliferation, migration and invasion in HCC. Furthermore, KCTD11 own the potential to judge prognosis of HCC patients. Overall, our findings provide a novel biomarker and molecule target in predicting, diagnosing and treating HCC.

## MATERIALS AND METHODS

### HCC tissue samples

The commercial tissue microarray containing forty-five cases of HCC tissues with paired peritumoral tissues (not contain survival information) was purchased from Alenabio (Cat. No. LV1504, Xi’an, China). Twenty-six cases of HCC tissues with matching peritumoral tissues were collected to test mRNA expression of KCTD11. A tissue microarray containing fifty-one cases of HCC tissues without paired peritumoral tissues was used, to perform survival analysis. Twenty cases of HCC tissues with paired peritumoral tissues were collected to perform LOH analysis. Except the commercial tissue microarray, all the tissues were from the First Affiliated Hospital, Zhejiang University School of Medicine, Zhejiang, China. Ethics approval was obtained from the ethics committee of this hospital.

### RT-PCR

A TRIzol Reagent (10296010, Thermo Fisher Scientific, USA) was used for total RNA extraction. TaKaRa PrimeScript™RT reagent Kit with gDNA Eraser (RR047A, TaKaRa) and Applied Biosystems 7500 Real Time PCR System were used to perform quantitative RT-PCR analysis. All the procedures and PCR parameters followed the instructions of these kits. Primers were synthesized by Sangon Biotech (Sangon Biotech, Shanghai, China). Primers for RT-PCR were as below: β-actin FW 5'-CACCATTGGCAATGAGCGGTTC-3', REV 5'- AGGTCTTTGCGGATGTCCACGT-3'; KCTD11 FW 5'-CACGCAGATGTAGATGTCAGCC-3', REV 5'-TCGGTGCAGAAAAGGTTGGCTC -3'.

### Immunohistochemistry (IHC)

Immunohistochemistry assay was performed as previously described [32]. A modified Histo-score (H-score) was performed to evaluate the protein expression. This score system consisted of percentage of positive cells (0%-5% 0; 5%-25% 1; 25%- 50% 2; 50%-75% 3; 75%-100% 4) and staining intensity (no 0; weak 1; moderate 2; or strong 3). The staining intensity score and percentage score were then multiplied to obtain a modified H-score.

### DNA extraction and LOH analysis

Genomic DNA was extracted by using the TIANamp Genomic DNA Kit (TIANGEN BIOTECH, Beijing, China). The primers was as the following: D17S960 FW 5′-TGATGCATATACATGCGTG-3′; D17S960REV 5′-TAGCGACTCTTCTGGCA-3′. PCR was performed by using 50 ng of genomic DNA, 0.2μM each primer, 5% DMSO, and platinum Taq DNA polymerase 1 U. PCR products were then separated in a denaturing 6% polyacrylamide gel (7M urea). The polyacrylamide was then performed silver stain to observe the lanes by using a Fast Silver Stain Kit (Beyotime, China). LOH positive was considered if an allele was absent or significant reduced (at least 50%) when compared to the corresponding normal alleles from the matching normal liver tissues.

### Cell culture

All cell lines were obtained from Cell Bank of Type Culture Collection of Chinese Academy of Sciences, Shanghai Institute of Cell Biology, Chinese Academy of Sciences. Cells were maintained in Minimum Essential Media (Cat. No. GNM-41500-S, Genom, China) with 10% fetal bovine serum(Moregate Biotech, Australia) and cultivated in a 37°C, 5% CO_2_ humidified incubator.

### Lentiviral construction and transfection

KCTD11 knock-down lentivirus (target sequence: TCCGACTAGACTCTGTCTT), ectopic expression lentivirus and matching negative control lentivirus (GFP expressed) were purchased from GeneChem (Shanghai, China). Transfection processes were according to the instruction provided by the manufacturer. The transfected cells were than screened in culture medium with puromycin (3μg/ml).

### SiRNA

The siRNA target sequences used in this study are as follows: CTGF siRNA, 5′-GAGAAGCAGAGCCGCCTGTGCATGGTCAG-3′; KCTD11 siRNA, 5′-TCCGGCACATCCTCAATTTCCTGAGGCTG-3′. The siRNAs were all synthesized by Origene (Origene, USA). The transfection concentration was 10 nM.

### Western blot

Western blot was performed as previously described [33]. To supplement, part of the western blot results were obtained by an image system (Universal Hood III, Bio-Rad, USA).

### Antibodies

Antibodies for western blot and immunohistochemistry: KCTD11(ab113423, abcam); KCTD11(GTX47451, Genetex); Flag(F1804, Sigma); p21(ab109520, abcam); Cyclin D1(2978, Cell Signaling Technology); Cyclin E1(4129, Cell Signaling Technology); CDK4(12790, Cell Signaling Technology); CDK6(3136, Cell Signaling Technology); CDK2(2546, Cell Signaling Technology); CLDN1(13050-1-AP, Proteintech); CTGF(ab6992, abcam); COL3A1(GTX27778, Genetex); ATP2A2(ab137020, abcam); CD24(GTX37755, Genetex); E-cadherin(ab40772, abcam); β-catenin(ab6302, abcam); MMP-2(1948-1, Epitomics); Slug(ab63568, abcam); GAPDH(DW880543, Hang Zhou Dawen Biotec, China); β-actin(DW9562, Hang Zhou Dawen Biotec, China) ; Hippo Signaling Antibody Sampler Kit (LATS1, p-MOB1, MST1, MST2, SAV1, p-Ser127-YAP, TAZ/YAP, #8579, Cell Signaling Technology); LATS2(5888, Cell Signaling Technology). Antibodies for co-immunoprecipitation: CTGF (ab6992, abcam); COL3A1 (GTX27778, Genetex); IgG (B900620, Proteintech).

### Cell viability assay

Cell viability was detected by Cell Counting Kit-8 (DOJINDO Laboratories, Kuma- moto, Japan). Seeded with cells (3×10^3^ per well for Huh7, 5×10^3^ per well for HCCLM3), 96-well plates were incubated in humidified incubator for 24h, 48h, 72h or 96h. With supernatant removed, every well was added by 100 μl medium and 10 μl CCK- 8, and then incubated for 1 h. The absorbance was detected at 450 nm on a microplate reader (BioTek, USA).

### Colony-forming assay

Colony-Forming Assay was performed as previously described [33]. To supplement, the 6-well plates were seeded with 3×10^3^ Huh7 cells per well or 5×10^3^ HCCLM3 cells per well.

### EDU assay

EDU assays were performed by using an EDU assay kit (Cat. No. C10310-1, Ribobio, China). Confocal dishes (2 cm) were plated with 5×10^4^ cells and incubated for 24 hours, the processes later were according to the instruction of this kit.

### Cell cycle assay

Cells (1.5×10^5^ per well) were seeded into 6-well plates. Serum starvation (24 hours) was adopted to perform synchronization. The cells were then incubated for 48 hours. After harvested, these cells were fixed by pre-cooled 75% ethanol at -20°C for 24 hours, and then resuspended by 300 μl DNA PREP Stain (Beckman Coulter, USA) and incubated in room temperature for 30 mins, away from light. The suspended cells were detected by BD LSR II (BD Biosciences, USA).

### Cell adhesion assay

Every well of 96-well plates was coated with 50 μl mixed liquor containing Matrigel (BD Biosciences, USA)/MEM at a 1:7 ratio for 2 hours and then coated with 50 μl MEM contained 10g/L BSA for an hour, in a 37°C humidified incubator. With the redundant liquid removed, 2×10^4^ cells were seeded into a well, and then cultured for 1 hour at 37°C in a in a 37°C, 5% CO_2_ incubator. The cells were then rinsed three times with PBS. The remaining cells were detected by Cell Counting Kit-8.

### Co-immunoprecipitation

Co-immunoprecipitation was performed as previously described [32].

### Cell migration and invasion assays

Millicell Cell Culture Inserts (24-well plate; 8μm pore size; Millipore, USA) were used to perform cell migration and invasion assays. For invasion assay, the inserts was coated with 35 μl mixed liquor containing Matrigel (BD Biosciences, USA)/MEM at a 1:7 ratio for 3 hours in a 37°C humidified incubator. For every insert, 3×10^4^ Huh7 cells or 5×10^4^ HCCLM3 cells in serum-free medium was seeded into the upper chamber. Culture medium containing 10% FBS was added into the lower chamber. The cells were incubated a 37°C, 5% CO_2_ incubator. Cells were incubated for 48 hours in migration assay and 72 hours in invasion assay. Cells on the lower surface of the membrane were stained by using Wright-Giemsa Stain Kit (Nanjing Jiancheng Bioengineering Institute, China).

### Nude mice xenograft and orthotopic model experiments

Animal experiments met the requirement of the guidelines of the National Institutes of Health (Guide for the Care and Use of Laboratory Animals, 2011). Cells were resuspended in 100 μl PBS and injected subcutaneously into the left flank of the mice (5×10^6^ cells per mouse for HCCLM3, and 2×10^6^ cells per mouse for Huh7). Tumor volume was calculated according to the formula as below: larger diameter × (smaller diameter)^2^/2. The subcutaneous tumors were removed for weigh and immunostaining in the 30th day. To establishe an orthotopic liver tumor model, two tumors from each group (the HCCLM3-GFP group and the HCCLM3-KCTD11 group) were cut into 1-2 mm^3^ cubes. Then, the cubes were implanted into the left liver lobe of other nude mice. After 8 weeks, the liver tissues and lung tissues were harvested. H&E staining was performed to analyze the tumor clusters in the lung tissues. Over 6 mice were performed in each group.

### Statistical analysis

Statistical analysis was performed using SPSS18.0 software. The data expressed as mean ± SD was assessed by two-tailed Student t-test. The association between KCTD11 expression and clinicopathologic characteristics was assessed by Chi-square test or Fisher exact test. Overall survival rate in Kaplan-Meier method was assessed by Log Rank test. P values ≤ 0.05 were considered significant.
